# An application of convolutional neural networks with salient features for relation classification

**DOI:** 10.1186/s12859-019-2808-3

**Published:** 2019-05-29

**Authors:** Zolzaya Dashdorj, Min Song

**Affiliations:** 1 Department of Library and Information Science, Yonsei University, 50, Yonsei-ro, Seodaemun-gu, Seoul, 120-749 Republic of Korea; 2 Department of Library and Information Science, Yonsei University, 50, Yonsei-ro, Seodaemun-gu, Seoul, 120-749 Republic of Korea

**Keywords:** Convolutional neural networks, Biomedical data analysis, Relation classification, Hyperparameter optimization, Deep learning

## Abstract

**Background:**

Due to the advent of deep learning, the increasing number of studies in the biomedical domain has attracted much interest in feature extraction and classification tasks. In this research, we seek the best combination of feature set and hyperparameter setting of deep learning algorithms for relation classification. To this end, we incorporate an entity and relation extraction tool, PKDE4J to extract biomedical features (i.e., biomedical entities, relations) for the relation classification. We compared the chosen Convolutional Neural Networks (CNN) based classification model with the most widely used learning algorithms.

**Results:**

Our CNN based classification model outperforms the most widely used supervised algorithms. We achieved a significant performance on binary classification with a weighted macro-average F1-score: 94.79% using pre-extracted relevant feature combinations. For multi-class classification, the weighted macro-average F1-score is estimated around 86.95%.

**Conclusions:**

Our results suggest that our proposed CNN based model using the not only single feature as the raw text of the sentences of biomedical literature, but also coupling with multiple and highlighted features extracted from the biomedical sentences could improve the classification performance significantly. We offer hyperparameter tuning and optimization approaches for our proposed model to obtain optimal hyperparameters of the models with the best performance.

## Background

**Fig. 1:**
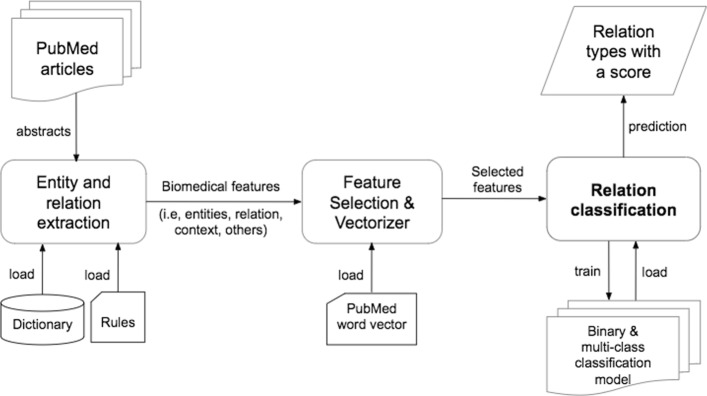
The overview of the PKDE4J architecture which includes our proposed relation classification module

An enormous amount of biomedical information is generated in terms of the results from biomedical experiments and a number of scientific literature describing the medication results, such as PubMed. The type of data is commonly represented in a form of unstructured text. Hence, a great interest for automated information extraction has been raised in the biomedicine and bioinformatics fields to support clinical needs and clinical decision making. Specifically, text mining techniques like Natural Language Processing and Computational Linguistics are frequently applied in the studies [1, 2] by adopting large annotate corpora (i.e., MEDLINE, DrugBank, DDI corpus, SemRep). Biological and biomolecules entities such as proteins and genes, chemical compound and drugs, disease names have been extracted [1, 3]. The classification of relation information between the bio-entities has been an emerging interest [2, 4, 5] to build a biomedical knowledge base. But in general, a feature extraction (i.e., entities and relation) is still a complicated task due to the complex structure of sentences and requires sophisticated methods of extracting syntactic, lexical and semantic features. Many studies [2, 5–9] propose a number of methods for improving feature extraction techniques coupled with categorization and classification. Thus, in this research, from the sentences of biomedical literature by coupling with a feature extraction tool, we intend to classify the relations between biomedical entities into defined relation types which have given and instructed by the domain experts. We use the not only raw text of the biomedical sentences, but also the highlighted and important features (entities, relations, and others) extracted from the sentences by a public knowledge discovery tool (PKDE4J) which has been developed in our previous research works [10, 11]. As the tool deals with an extensive type of entity corpora, we are interested in recognizing the cross-domain relationship of the entities and attempted to build a common classification framework which will be an extension of the PKDE4J. The overview of the PKDE4J tool architecture is presented in Fig. 1 and the tool can extract multiple types of biomedical entities, relations, and relevant contextual information comprehensibly with its extensible and flexible architecture. In recent studies, performance on text classification has been achieved better by incorporating deep learning tools [12–14]. Hence, we incorporate CNN in our relation classification task. With the use of correctly annotated relation types by a number of domain experts in the biology domain, we evaluate our proposed classification model and compared with widely used supervised learning models. Our findings indicate that our proposed relation classification approach better predicts a possible relation type with a certain accuracy solely using the raw text of the sentences. By coupling with pre-extracted features from the raw text, the model performance increases with a smaller degree. In fact, CNN based models require more practical knowledge to configure the model architecture with regard to the performance [15] and to set the hyperparameters for the best optimization [16]. To resolve this issue, we conduct an extensive evaluation such as hyperparameter tuning and optimization in order to explore the reasonable ranges for the sensitive hyperparameters of the classification model. Our research outcomes can provide a benefit to a variety of applications, including recommendation systems in bioinformatics and biomedicine fields.

### Related works

Many approaches to relation extraction are developed in [10, 17] using the recent advance of the text mining and NLP tool. Traditionally, structure features (e.g., the shortest dependency path between nominals) are used to solve this problem [18]. However, the word features were not enough to capture the structure of the biomedical text and many issues are still remaining. There is an increasing interest in supervised relation extraction and classification [4, 19–22] for improving the relations. The relation classification aims at the prediction of further relations between the predefined type of entities in the corpus. The researchers in this field, mostly employ supervised multi classifiers depending on the input to the classifiers. [19] divides the relation classification approaches into feature-based (i.e., Max Entropy, SVM) [23, 24], tree kernel-based [8, 25, 26] and composite kernel-based [9]. In recent studies, Deep Learning Networks (DLN) have been greatly applied to the relation classification problems [5–7]. This approach is becoming increasingly effective [7, 12–14, 27]. A convolutional deep neural network is used by Zeng et al. [5] over the whole sentence combined with some lexical features in order to extract lexical and sentence level features. Words position as feature vectors were obtained with the corresponding word embedding and the combination of learned lexical and sentence level features was effective for relation classification. Recurrent Neural Network is constructed with the combination of matrix-vector representation which presented by Socher et al.[6] in order to learn compositional vector representation for phrases and sentences of arbitrary syntactic type and length. This model has shown better performance in the classification of semantic relationships between nouns in a sentence. Yu et al.[7] proposed a compositional model which extracts sentence level and substructure embeddings from word embeddings using global information. The embedding approach combined with the log-quadratic model outperforms the comparative approaches. SemEval-2010 Task 8 [2] has proposed a multi-way classification for common nominals. In a similar fashion, our research encounters a cause-effect relationship between the entities. [13, 14, 27] studied the effective use of CNN for sentence-level classification tasks with the use of pre-trained word vectors and the models achieve remarkably strong results. [15] extended the effectiveness and analysis of the CNN models by considering extensive model variants (i.e., filter widths, k-max pooling, and different word vectors). Hence, in a similar fashion, we conduct our classification experiment on the manually labeled dataset. In learning networks, it is important to emphasize hyperparameters at an optimal configuration that could improve the classification performance with a certain accuracy. A number of studies have been conducted on improving classification tasks by hyperparameter tuning and optimization [16, 28]. The study result shows that compared to the grid search optimization, the random search optimization algorithm finds models better and effectively with a less computation time and few hyperparameter candidates.

## Methods

### Data pre-processing

**Fig. 2:**
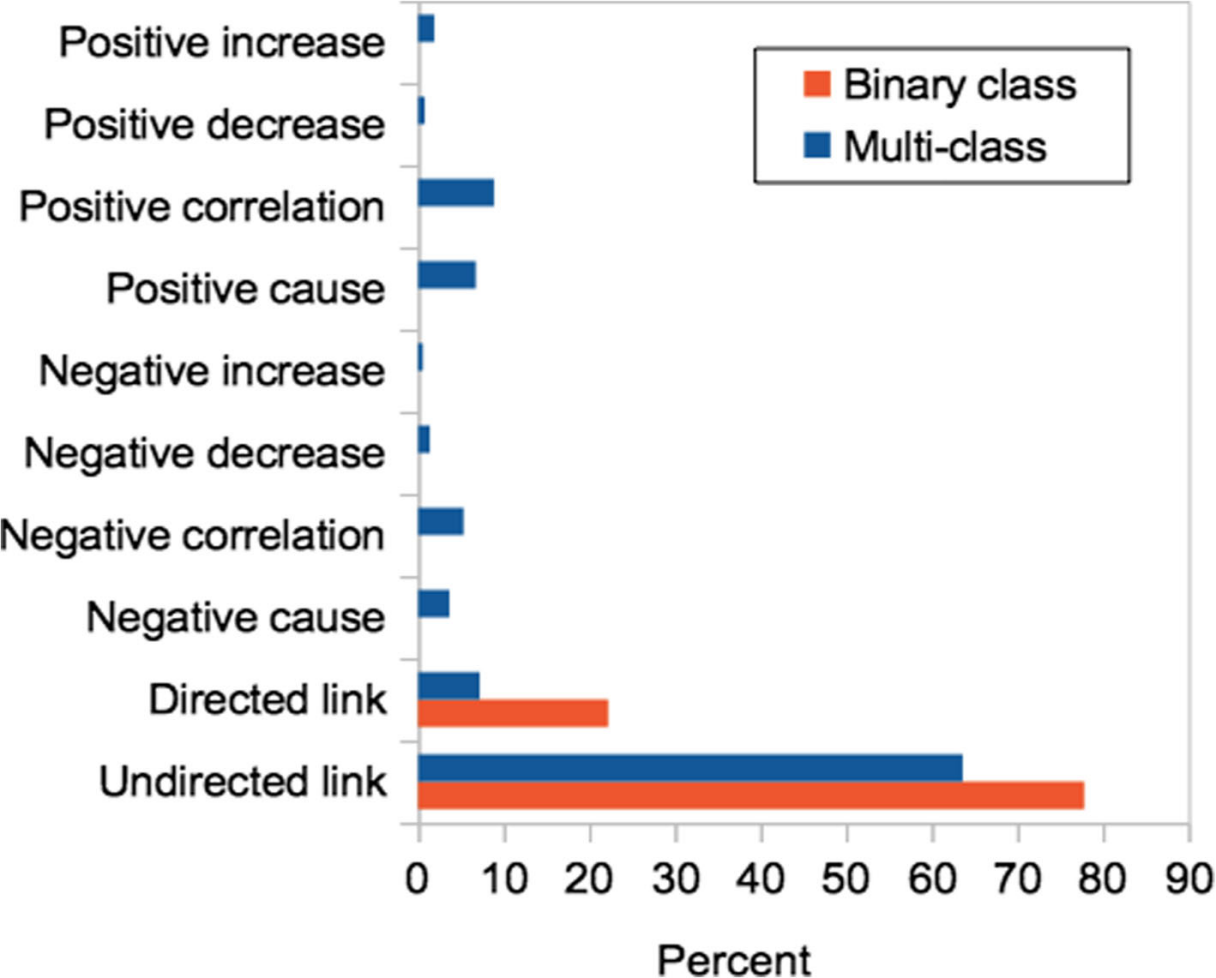
The distribution of the labeled dataset for binary and multi-class classification

In this study, we sampled the 1,156 number of PubMed articles and extracted about 7,143 important biomedical feature records for our relation classification model by using a public knowledge discovery tool, PKDE4J [10, 11] in order to feed our proposed classification model. The tool extracts totally 15 different features for each sentence of given biomedical literature, which are explained in Table. 1. For instance, a given sentence “Dehydroepiandrosterone sulfate increases hepatic ubiquinone-9 in male F-344 rats”, the feature “Dehydroepiandrosterone sulfate” is extracted as a compound type of entity, the feature “ubiquinone-9” is also as a compound type of entity that related through a relation verb, “increases”. The details of the features are explained in Table. 1. In the early stage of our classification model, as no resource is yet available to evaluate the functionalities of our model, we decided to build our own labeled evaluation set: we labeled resulting 2,167 instances of the feature records for the purpose of training and testing. To limit real-world problems, such relation types were labeled carefully with the help of domain experts in the field of biomedicine. We also clean and discard feature records containing irregular or irrelevant biomedical entities and the relation. To this end, the relation between biomedical entities for each record is manually labeled into two types of relations that one describing binary classification and the other one describing multi-class classification. The distribution of the labeled datasets for the two classifications we enriched for our research is depicted in Fig. 2. However, our training data is imbalanced, we attempt to improve the classification performance in biomedical data records.

**Table 1:** The description of features with an example that extracted from a biomedical sentence by an entity and relation extraction tool, PKDE4J

No	Biomedical features	Description	Example feature record
1	ID	Identifier number of an article	15248468
2	Sent ID	Sentence id of a sentence	0
		in the abstract of an article	
3	Entity-L	Entity on the left side	dehydroepiandrosterone sulfate
4	Type-L	Type of the entity on the left side	COMPOUND
5	Context-L	Context of the entity on the left side	NA;
6	Entity-R	Entity on the right side	ubiquinone-9
7	Type-R	Type of the entity on the right side	COMPOUND
8	Context-R	Context of the entity on the right side	NA
9	Negation	Negativeness of the relation	POSITIVE
10	Tense	Tense of the relation	ACTIVE
11	Verb	Verb of the relation	increase
12	Relation	Reference word of the relation	LOCATION_OF
13	Context level	Level of the context in the relation	level=0
14	Verb phrase	Verb phrase	increases hepatic ubiquinone-9 in
			male F-344
15	Sentence	Raw text of a sentence	Dehydroepiandrosterone sulfate
			increases hepatic ubiquinone-9
			in male F-344 rats

### Pre-trained word vector

We use a pre-trained word vector model as proposed in [3] for the proposed classification model. The word vector model is 200 dimensional in binary format and induced from PubMed abstracts^1^ of over 5 million tokens using word2vec^2^. The skip-gram model with a window size of 5, hierarchical softmax training, and a frequent word subsampling threshold of 0.001 is applied to the word vector.

### Convolutional neural networks based classification model

**Fig. 3:**
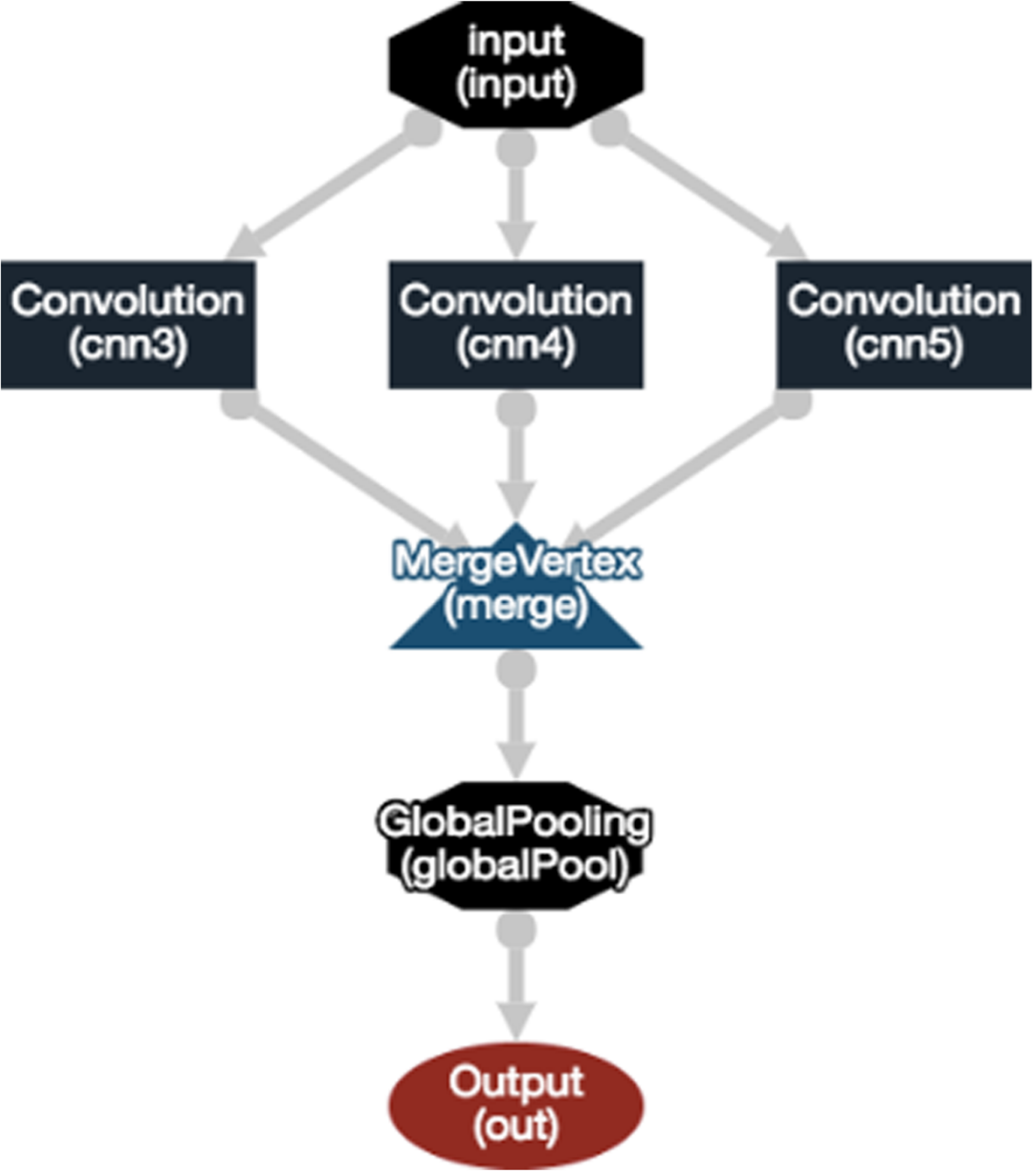
The architecture of our proposed CNN classification with multiple convolutional layers

We present our proposed model employing CNN which classifies the relations between biomedical entities. We set our model architecture based on widely used strategies in a similar fashion which proposed in [12, 13, 15]. The architecture of our CNN based classification model is visualized in Fig. 3 which consists of three convolutional layers followed by a global pooling layer. We firstly feed our model with the pre-generated PubMed word vector for vectorizing and reducing dimensions of the training and test sets that we use in our research. Given the features in each record of *n* number of records, we group the features based on their importance for the classification task and concatenated into a single text donated by *s*_*im*_ which was limited up to 256 character length *m*. The PubMed word vector induces the texts $s_{nm} \in \mathbb {R}^{nk}$ into an embedding vector matrix denoted by *x*_*nk*_ in which the row *x*_*n*_ is the embedding vector representation of *s*_*n*_. With linear filters, we perform convolution on the embedding vector matrix. The filter width is equal to the dimension *k* of the embedding vector. The height of the filter is a number of adjacent rows considered jointly and referred to the region size *h*. The convolutional weight matrix *w* with the region size is obtained by the convolution operator repeatedly in order to produce a feature map matrix $A \in \mathbb {R}^{n-h+1}$. The model uses multiple filters (i.e., three filters) with varying window sizes to obtain multiple feature maps. The dimensionality of the feature map generated by each filter will vary as a function of the sentence length and the filter region size. We denote the feature map by *A*_*i*_=*f*(*w*×*x*_*i*:*i*+*h*−1_+*b*), where *i*=1...*n*−*h*+1 and *b* is a bias term and *f* is an activation function as non-linear function to each *A*_*i*_. We then applied a max pooling function [12, 29] over the feature maps and take the maximum scalar from each feature map in order to capture the most important feature for each feature map. The outputs are concatenated into a top-level feature vector in a fixed length and passed to a fully connected softmax layer with a regularization, dropout [30, 31] in order to generate the final classification output. The final output is the probability distribution over labels. We use a categorical cross-entropy loss to minimize the reasonable training objective.

## Results

To estimate the effectiveness of the supervised learning models in relation classification task, we demonstrate extensive experiments on biomedical entities and their existing relation types extracted from biomedical articles by PKDE4J by incorporating deep learning tools 3. Although, the relation classification performance is strongly affected by the quality of the extracted features. We employ a CNN based classification model in order to study baseline and compare the results with other competitive and conventional learning models such as Support Vector Machine (SVM), Stochastic Gradient Descent (SGD), Naive Bayes (NB), Logistic Regression (LR), Random Forest (RF) and K-nearest neighbor (KNN). We then evaluate our proposed CNN based classification models by hyperparameter tuning and optimization approaches for measuring the effectiveness of the different settings of hyperparameters in our proposed models and to obtain the best-performed model with an optimal configuration.

### Training dataset

**Fig. 4:**
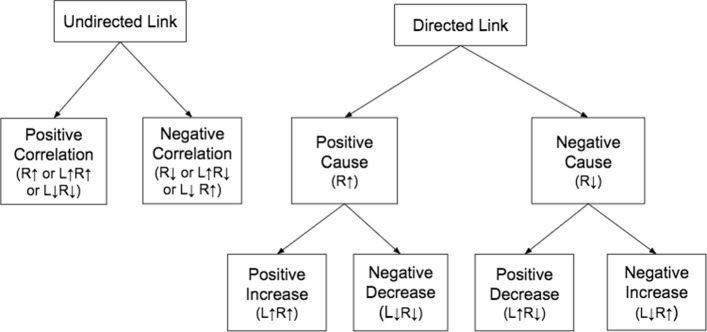
The hierarchy of relation classification: The right side entity is represented by R and the left side entity by L. *↑* represents a growing state and *↓* represents a declining state of an entity that affected by the relation verb

We describe here a methodology of how we manually label the biomedical feature records which extracted by PKDE4J for building a training dataset, with the help of domain experts in biomedicine field. However, our classification types were given without a hierarchy and pre-defined by domain experts that are illustrated in Fig. 4. We divide those classification types into two models: a coarse-grained—binary classification—and a fine-grained—multi-class (10 classes) classification—.

In binary classification, we define a relation type, as *Directed Link* if one bio-entity causes another bio-entity directly in passive or active form through a relation verb without any known or given the effect of a cause on the bio-entities. Otherwise, *Undirected Link* is defined as a relation type that one bio-entity associate with another bio-entity indirectly with no cause-effect on the relation and the bio-entities. In our context, a relation verb describes the relation between bio-entities and this relation can be a cause or no cause. The instances of relation verb are as following: prohibit, associate, induce, increase. We further specify the relation types into fine-grained types which basically describe a cause-effect relation. To this end, we encounter a trigger word to understand the effect on the bio-entities. A trigger word can be either a noun or a verb. The examples of a trigger word that describes the effect on a bio-entity are as following: activation, increased, active, reduction.

The relation types in multi-class classification—*Positive Cause* and *Negative Cause*—are defined if a bio-entity causes another bio-entity directly through a relation verb for a growing effect or a declining effect, respectively. In case the trigger word for a bio-entity or bio-entities is given, the relation type is encountered depending on the effect on the bio-entity or the bio-entities as *Positive Increase* or *Positive Decrease* or *Negative Increase* or *Negative Decrease*. In a similar fashion, *Positive Correlation* and *Negative Correlation* are defined but the bio-entities are related indirectly by a relation verb. For instance, in a given feature record extracted by PKDE4J from the bio-medical sentences of literature as presented in Table 1, a relation verb between the two biomedical entities (*dehydroepiandrosterone sulfate* and *ubiquinone-9*) is extracted as *increases*. We define this relation type as *Directed Link* in binary classification and *Positive Cause* in multi-class classification.

### Convolutional neural networks based classification model performance

In this section, we study our proposed classification model that configured with a common architecture and common hyperparameters based on a manual search, as there is no official development dataset and preliminary studies to evaluate our model. The hyperparameters are set as the good performing CNN based architecture in [13, 15] which are word dimension of 200, filter kernel size (w) of 3, 4, 5 in the convolutional layers with 300 feature maps each, update rule as Adadelta, transfer function as rectified linear, pooling dropout rate (p) of 0.5 as common, l2 constraint (s) of 0.0001, and mini-batch size of 32 with up to 500 epochs. We use global max pooling in the output layer. To avoid the network from over-fitting, we apply an early stopping of the network: iterate the number of training epochs and stop the iteration if the network outperforms the previous best model on the validation set. We conduct 5 fold cross-validation in this experiment for binary and multi-class classification.

However, our datasets are imbalanced but we elaborated balanced per class data in training and validation sets for the cross-validation and attempted to estimate the performance of our proposed model in order to compare it with the other supervised algorithms. In a classification task, feature selection plays an important role in feeding the model well. Our main features are the sentences of biomedical literature, but a sentence is a raw data, very noisy and complex in terms of the hidden sentence structure. Thus, we use the not only raw text of sentences, but also pre-extracted features from sentences are considered in this study. The features are extracted by PKDE4J from the sentences of biomedical literature. Five groups of important and most relevant features are manually proposed as stated in Table 2 with the help of domain experts.

**Table 2:** Entity-Relation Group based on pre-extracted features in sentence level and the raw text of sentences

Group No	Type	Selected features concatenated with a order
Group 1	Feature	Entity-L, Entity-R, Verb
Group 2	Feature	Entity-L, Entity-R, Negation, Tense, Verb, Relation
Group 3	Feature	Entity-L, Type-L, Context-L, Entity-R, Type-R, Context-R,
		Negation,Tense, Verb, Relation, Context level, Verb phrase
Group 4	Feature and	Entity-L, Type-L, Context-L, Entity-R, Type-R, Context-R,
	Sentence	Negation, Tense, Verb, Relation, Context level, Verb phrase,
		Raw text of a sentence
Group 5	Sentence	Raw text of a sentence

The first three groups are pre-extracted features in different manners for feeding the model: Group 1 consists of a few numbers of pre-extracted features; Group 2 includes very relevant pre-extracted features; Group 3 consists of an extensive number of pre-extracted features. The other two groups are a combination of pre-extracted features and the raw text of sentences for Group 4, and the raw text of sentences solely as a single feature for Group 5. In order to feed our CNN based classification model, we sequentially concatenated the features in each group to transform into plain text. Then the plain text is vectorized for training and validating. For example, for Group 1, the three pre-extracted features (entities, and relation) are sequentially concatenated into a plain text as “dehydroepiandrosterone sulfate ubiquinone-9 increase”, as stated in Table 1. However, the order of the features is important to feed the model, but in this research, we do not study the importance of the order of pre-extracted features explicitly as the hidden layers in CNN is able to obtain the significant convolutional features via neurons, and the order of pre-extracted features is taken into account as stated in Table 2.

For each combination, we estimated accuracy, precision, recall, and F1-score, respectively for our CNN based binary and multi-class classification models as reported in Tables 3 and 4 as well as other conventional supervised algorithms. For precision, recall, and F1-score, we apply a weighted (equally) macro-average metric method. However, the maximum performance on binary classification was achieved as F1-score of 95.18% for Group 3 in which an extensive amount of pre-extracted features were used. But, in terms of the good recall, the best performance was observed on Group 2, around F1-score of 94.79% at the maximum that uses only pre-extracted features; followed by Group 3. The highest overall average F1-score among the groups, was estimated on Group 2 which is around 88.29%. This approach improves the models by 1-2% which use the combination of pre-extracted features and the raw text of a sentence as in Group 4 or using solely the raw text of a sentence as in Group 5. We suggest that using pre-extracted features from the biomedical sentences of literature improves relation classification at a certain accuracy instead of solely using the raw text of the biomedical sentences.

**Table 3:** The performance (weighted macro-average metric) of CNN based binary classification model on validation set

Group No	Accuracy	Precision	Recall	F1 score
	Max/Ave/Min	Max/Ave/Min	Max/Ave/Min	Max/Ave/Min
Group 1	89.81/77.19/57.27	72.92/56.85/45.22	51.85/50.65/49.62	94.63/86.26/71.14
Group 2	90.28/82.42/72.29	77.10/72.45/69.55	70.93/64.45/58.61	94.79/88.29/76.83
Group 3	90.97/81.45/69.05	80.40/72.36/66.74	66.90/62.11/58.32	95.18/87.90/75.64
Group 4	90.28/81.22/68.13	79.69/71.30/66.96	64.66/59.67/53.13	94.85/88.10/76.37
Group 5	89.58/78.4/59.35	87.35/65.23/57.90	60.08/53.81/50.92	94.48/86.94/72.59

**Table 4:** The performance (weighted macro-average metric) of CNN based multi-class classification model on validation set

Group No	Accuracy	Precision	Recall	F1 score
	Max/Ave/Min	Max/Ave/Min	Max/Ave/Min	Max/Ave/Min
Group 1	77.74/68.69/60.74	88.54/49.78/26.35	11.11/10.5/10.25	47.70/36.3/21.44
Group 2	76.67/63.68/44.24	76.67/55.67/20.23	11.11/10.22/10.00	86.80/67.19/25.18
Group 3	76.91/63.77/44.47	76.91/64.50/48.15	11.11/10.26/10.00	86.95/69.33/21.82
Group 4	76.91/63.77/44.24	76.91/63.77/44.24	11.11/10.22/10.00	86.95/77.26/61.34
Group 5	76.91/63.77/44.24	76.91/63.77/44.24	11.11/10.22/10.00	86.95/77.26/61.34

**Fig. 5:**
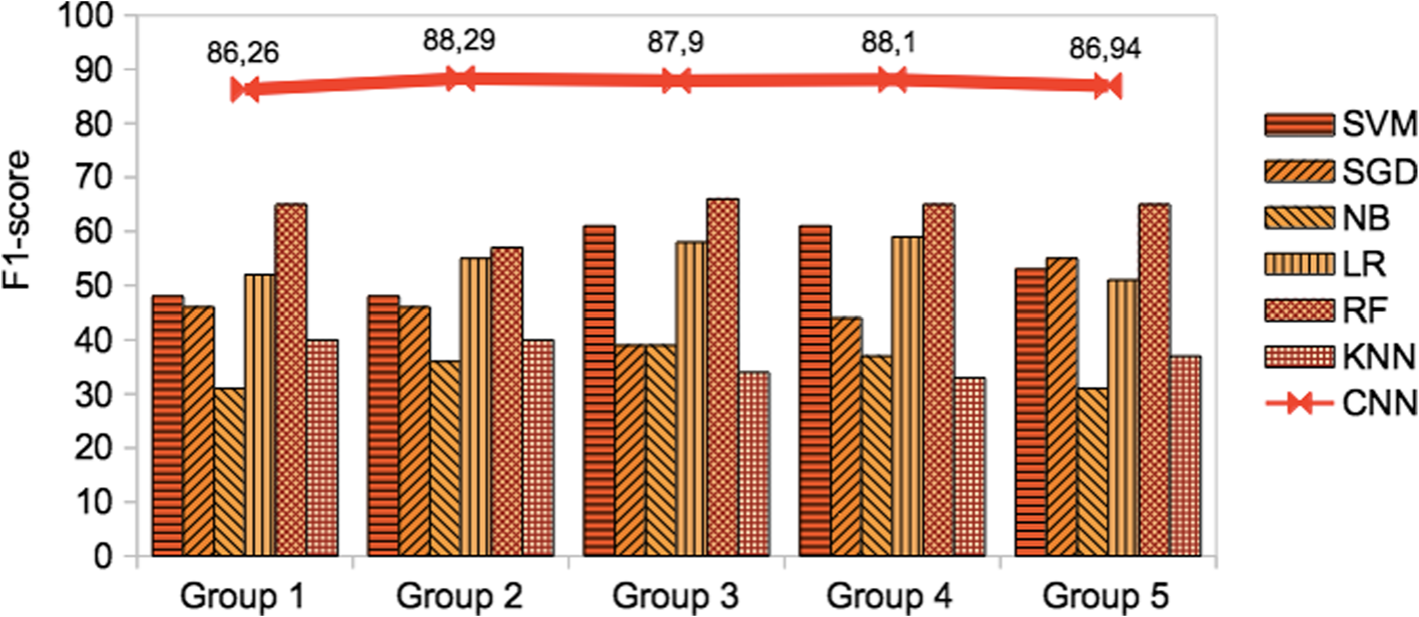
The performance (weighted macro-average F1-score) of our proposed CNN based binary classification model

**Fig. 6:**
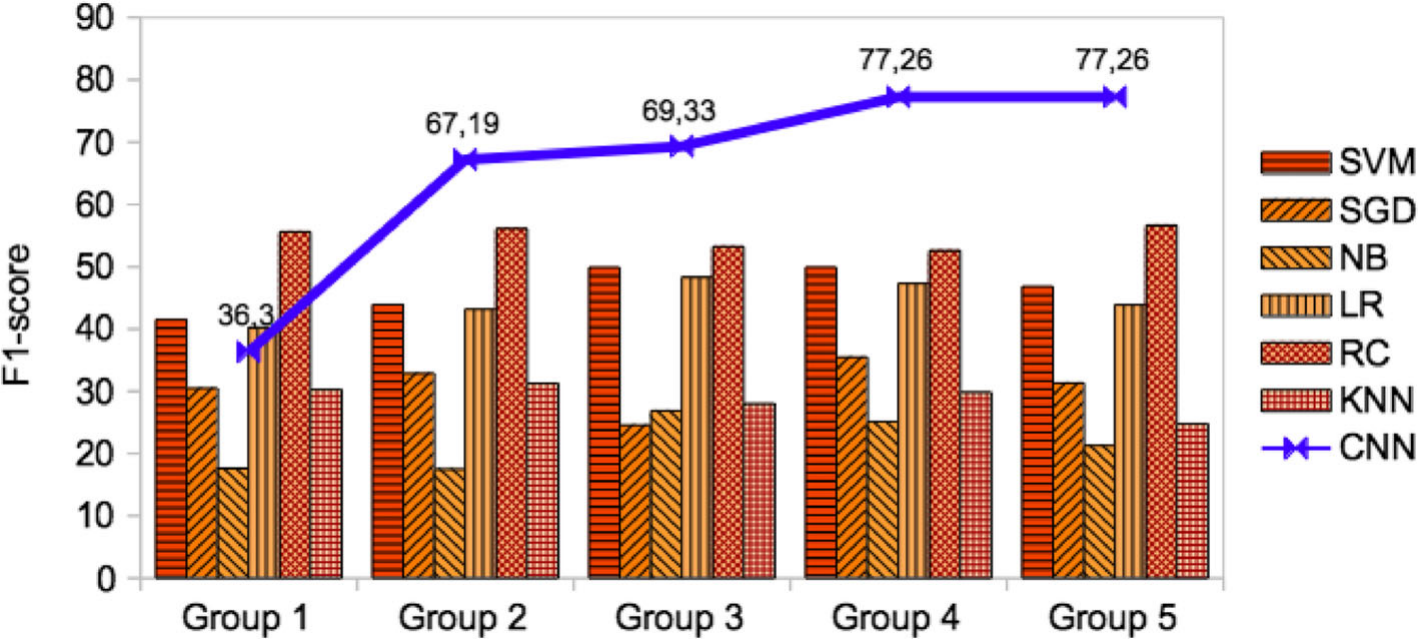
The performance (weighted macro-average F1-score) of our proposed CNN based multi-class classification model

For multi-classification, the maximum achievement of the weighted macro-average F1-score was also estimated on Group 4 and Group 5, around 86.95%. But the overall recall for each group was relatively low around 10.2% that requires further improvement on multi-class classification by considering a large number of training data per class in order to increase the per class prediction performance. In contrast to the results in binary classification, the overall average F1-score on Group 4 and Group 5 were better than the other groups in which the raw text of a sentence or the combined with the important features were used respectively. Because Group 4 and Group 5 include both the relation verb and the trigger word in addition to the bio-medical important features that are very useful to recognize a cause-effect relationship in multi-class classification. In the future, we compare the macro-average metric with the micro-average metric. However, the current version of PKDE4J that we use in this research is not able to extract a trigger word due to the restricted and heuristic feature extraction rules set in our experiment. Thus, we further extend PKDE4J by coping with the trigger word extraction as an additional feature for multi-class classification. Therefore, the overall average estimation over groups was compared with the results by using different supervised algorithms for the two classifications that described in Figs. 5 and 6, respectively. The CNN based model significantly outperforms the other supervised learning algorithms with a good prediction performance. Thus, the results justify the advantages of CNN based model over conventional baseline methods. We also noted for widely used learning algorithms that coupling with feature extraction improves the classification performance to some extent in a similar fashion to the CNN approach. However, our multi-class CNN based model performance was largely affected by the number of training data sampled for each class. But we further propose a different sampling method for collecting an extensive amount of equally distributed and well balanced labeled data over classes.

### Hyperparameter tuning

**Fig. 7:**
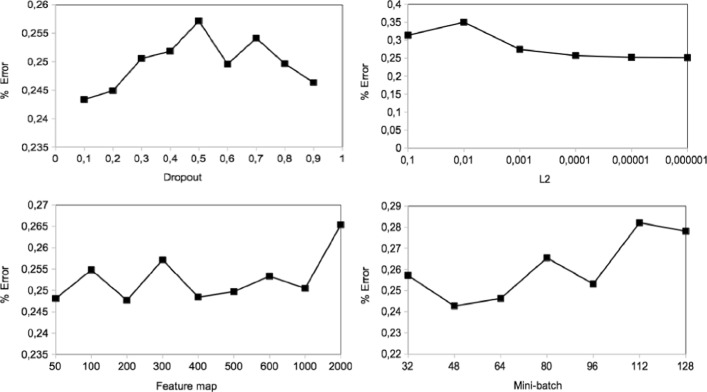
Hyperparameters tuning on binary classification: hyperparameters effect vs loss function value

In this section, we experimentally study the effects of the hyperparameters on the performance of our proposed method to estimate how much the model performance could be improved. The common regularization approaches in the CNN based method are a dropout, batch size, l2 norm constraint, and convolutional feature map size to avoid under-fitting and over-fitting during the training. Due to the limited number of training dataset for multi-class classification, tuning with different hyperparameters rates were not effective in improving the performance comparison. In this experiment, we extend the analysis of the binary classification as the results show a significant compared the multi-class classification. To compare the results, we use the best performing cross-validation set that is the dataset of Group 2 with F1-score of 94.79%. Figure 7 presents the effect of dropout, regularization, convolutional feature map, and mini-batch, respectively.

We first studied the dropout rate applying it for the pooling layer. The dropout rate varies from 0.1 to 0.9 and 0.5 as default. It is a probability of retaining a unit in the max pooling layer. We used a common dropout rate of 0.5 in our CNN based model. The performance results are shown in Fig. 7. We found out that the dropout at 0.1 in order words 90% of neurons activation decreases the classification error by 1.38%. We notice that the dropout helps little, and small dropout rate causes overfitting, large dropout rate dramatically hurts performance. For instance, F1-score of 95.05% was observed at dropout rate 0.7 that is a better performance than our proposed CNN based estimation.

We, therefore, studied the regularization norms of our binary classification on a range of values. Regularization is useful to avoid overfitting during the model training and penalize large network weights. The common values for regularization are 1e-3 to 1e-6. In Fig. 7, the regularization decreases slowly over the regularization which decreases. For example, the classification error was at *l*2=10^−6^ reduced by 0.58% at *l*2=10^−4^ compared to our proposed CNN based estimation.

We further extended the hyperparameter tuning experiment by demonstrating the different size of feature maps in the convolutional layers, as presented in Fig. 7. This study highlights that the feature map at 50 sizes followed by 200, 400 could be the optimal number for this classification and the map size at 50 can reduce our CNN based estimation error by 0.9%. A similar to dropout rate exploration experiment, feature map helps a little improvement on the performance. The error rate increases when the feature map size increases. Thus, the optimal range of the feature map size is fallen between 50 and 1000.

The sensibility of the model was studied with the mini batches in the range of 32-128 being common in terms of GPU. The mini batch is the number of training data used in one iteration. The ideal mini-batch size varies depending on the total number of the training set. Because for each iteration, the gradient of the loss function is estimated and the network parameters are updated. Figure 7 shows the classification error (loss function value) over the different mini-batch sizes. The model using a mini-batch size at 48 followed by 64 is beneficial for this classification. However, the effect of tuning n is relatively varied, but the overall classification error difference was relatively lower. The error rate was increased when the mini-batch size was increased. Thus, the optimal range for the mini-batch is considered in the between 32 and 96. Moreover, less important regularizers would be our further interest in exploring the performance improvement, such as activation function, filter region size, word vectors.

### Hyperparameters optimization

The hyperparameter tunning experiment has shown a great effect in model regularization, but that approach is often computationally intensive. To make the computation at low cost, or to get a suggestion on setting hyperparameters for a learning algorithm at the earliest configuration in case of no pre-defined studies, we demonstrate hyperparameter optimization on our proposed CNN based classification. Thus, we use common and more practical optimization techniques (Random search, Grid search) that proposed in [18, 32] with the same CNN architecture as our proposed CNN based classification in order to obtain optimal hyperparameters. But the experiment is focused on binary classification by employing the best performing cross-validation dataset on Group 2 which was significant to train the model well. We first set hyperparameters as following: dropout=0.5 (default), learning rate {0.00001, 0.001, 0.1}, regularization {0.00001, 0.001, 0.1}, and activation function as ReLU, LeakReLU, Sigmoid and Tanh with updater as AdaMax in the optimization algorithm of Stochastic Gradient Descent. The performance estimation by random and grid search optimization was compared in Table 5 including the hyperparameters of one of the best models suggested by those search optimization algorithms. For precision, recall and F1-score, we applied a weighted (equally) macro-average metric method.

**Table 5:** The optimization performance (weighted macro-average metric) in binary classification

Optimization	Random search	Grid search
Execution time	1150.446s	1688.907s
Accuracy	90.53%	87.30%
Precision	90.53%	60.07%
Recall	50.00%	58.04%
F1-score	90.53%	93.06%
Suggested	learning rate=0.001; l2=1.0E-5;	learning rate=0.1; l2=1.0E-5;
hyperparameters	beta1=0.001; beta2=0.001;	beta1=0.001; beta2=0.001;
	epsilon=0.001; activation=ReLU;	epsilon=0.001; activation=ReLU;

We highlight that the optimization algorithms achieve approximately equal performance to our CNN based approach with a common architecture. In the future, we also compare the macro-average metric with the micro-average metric due to the class imbalance. But the grid search performance was achieved 2.53% better than the random search. But the results suggest that the random search optimization performs more efficient than grid search optimization in terms of the execution time, as the findings observed in [18, 32]. In contrast, hyperparameter tuning could produce relatively better performance precisely for every value of different rage of parameters’ space at an expensive cost. However, the random search might be more efficient and requires less computational time as all hyperparameters are not equally important in the classification. But it could suggest us a hint for optimal hyperparameters by obtaining one of the best performing models if one does not have enough experience in the study. The experiment can be extended with a computationally expensive cost by incorporating more values on the hyperparameter space in the feature map and kernels in convolutional layers. We further try to enhance our classification performance by demonstrating a Bayesian optimization [28].

## Conclusions

In this paper, we study relation classification with a variety of different feature combinations that extracted by a feature extraction tool of PKDE4J from PubMed articles. We offer a CNN based classification model with a state-of-the-art architecture for predicting a type of relation between bio-entities that included in the sentences of biomedical literature. We conduct 5 fold cross-validation. Our proposed CNN based models outperform most widely used supervised algorithms. We achieved a significant recall and precision on binary classification with a weighted macro-average F1-score: 94.79% at the maximum using pre-extracted relevant feature combinations and the overall average F1-score was estimated 88.29% that was an improvement of 1.4% more than the model solely using a single feature as the raw text of the sentences of biomedical literature. Thus, our finding highlights that an appropriate feature extraction is essential to improve the model performance significantly instead of using raw data as the raw text of the biomedical sentences of literature due to the complex sentence structure as well as the various features with different weight. But for multi-class classification, the weighted macro-average F1-score of 86.95% at the maximum was estimated with the very low recall of 10.2%. The overall average F1-score was estimated around 77.26%. Therefore, the demonstration of hyperparameters tuning and optimization outperform our proposed CNN model architecture to some extent by considering dropout, regularization and mini-batch size. But random search optimization could produce rather equal performance at low cost. As a follow-up study, we plan to improve the relation extraction task based on SemRep, DDIExtraction-2013 Shared Task, SemEval-2010 Task 8 [2, 4, 33] by employing deep neural network. We also consider trigger words as additional features for multi-class classification task to analyze the effect on the bio-entities. We then favorably explore the best combination of features and hyperparameter selections for multi-class classification on the increased number of training data per class.

## Abbreviations

CNN: Convolutional neural networks
DLN: Deep learning networks
KNN: K-nearest neighbor
LR: Logistic regression
NB: Naive bayes
PKDE4J: Public knowledge discovery tool
RF: Random forest
SGD: Stochastic gradient descent
SVM: Support vector machine

## Footnotes

1 http://evexdb.org/pmresources/ngrams/PubMed

2 https://code.google.com/archive/p/word2vec

3 https://deeplearning4j.org
